# Abstracts and Gleanings

**Published:** 1879-12-20

**Authors:** 


### ABSTRACTS AND GLEANINGS.

### *EXTRACTS FROM SERVICE OF WM. GOODELL, M. D.*

[Reported for the MEDICAL RECORD.]

**Laceration of the Perinuem.**—Dr. Goodell advises the immediate operation, which he has found to be very successful in incomplete lacerations. In complete laceration it is not as successful as the secondary operation. In the primary operation, in order to put in the stitches accurately, Dr. Goodell recommends that ether be given, and that a sponge be placed high up in the vagina to stop the flow of the lochia, which embarrasses the operator. The stitches are applied as in the secondary operation, and merely twisted together. In the secondary operation, if the sphincter ani is involved, he always imbeds the first two stitches. On the eighth day all the stitches are removed, except the lowest. The fæces are then softened by an injection of warm sweet oil, and the bowels are moved twelve hours later by an ounce-dose of castor oil, aided, if necessary, by an injection. After the bowels have been emptied, the remaining stitch is removed.

**Vaginitis.**—Non-specific and acute cases of vaginitis, he treats by such hot and emolient injections as flaxseed, or slippery-elm bark tea, to which laudanum has been added; the solution which he usually employs contains laudanum f. ℥ j. to Oij. of flaxseed. When the inflammation has subsided, vaginal suppositories, containing five grains of iodoform, are ordered twice or thrice daily. In the chronic forms of this complaint, suppositories of tannin, or of iodoform, or long tampons of absorbent cotton, are employed, which have been dipped in astringent solutions of acetate of lead and zinc to which laudanum has been added.

**Carbuncle of the Urethra.**—In timid women who refuse to submit to an operation, he either mummifies the growth with crystallized carbolic acid melted down by heat, or destroys it by applications of chromic acid made with the utmost care, by means of a match whittled down to a point. The excess of acid is afterward neutralized by means of injections of a strong solution of the bicarbonate of sodium. If an operation is permitted, he cuts off the growth with a pair of scissors curved on the flat, and sears the wound with a hot iron wire, or with Paquelin's thermo-cautery. He advises the use of an alcohol lamp for heating the wire, because, when an ordinary light is used, the impression upon the operator's retina, made by the bright flame, so obscures his vision that the wire grows cool before he can clearly see the point where the application is to be made. He hastens the healing of the cauterized surface by occasional applications of carbolic acid, or by dusting it with iodoform.

**Cancer of the Cervix.**—Whenever practicable, the whole cervix is removed by either the hot or the cold wire. If this cannot be done, he removes the malignant growth by scraping or by means of the gouge-forceps, and the surface is subsequently charred with the thermocautery. This radical treatment is reinforced by subsequent applications of the ethylate of sodium. In these operations upon the cervix, Dr. Goodell finds that injections of ordinary vinegar form an excellent means of controlling any embarrassing bleeding. By these means he has succeeded in curing several cases.

**Cystitis in the Female.**—Transient cystitis, dependent upon obscure causes, is treated by rectal suppositories containing one grain each of the aqueous extract of opium and of the extract of belladonna. Hysterical cases generally yield to "massage" and electricity. In obstinate cases, Dr. Goodell warmly advocates the dilatation of the urethra throughout its whole extent by the introduction of the forefinger. In the therapeutical treatment of this troublesome disorder atropia is the most efficient remedy, and it may be combined with alkalies or acids, according to the condition of the urine. Injections of a two-grain solution of quinia into the bladder, together with large doses of the same by mouth, will often improve the condition of the patient. In very bad cases, perhaps the most efficient injection is one of the nitrate of silver, beginning with weak solutions and increasing their strength daily until twenty grains to the ounce solutions are tolerated. These strong solutions should not be allowed to remain in the bladder longer than ten or fifteen seconds. All malpositions of the womb must, of course, be rectified, especially if they have any bearing on the disease.

**Ovariotomy.**—In six cases of ovariotomy performed within the past nine months, with but one death, the following procedure was invariably adopted: A five per cent. solution of carbolic acid was used in the spray, and all instruments and sponges were immersed in a solution of the same strength. The pedicle was treated by the intra-peritoneal method, being transfixed, tied, and dropped within the abdominal cavity. The peritoneum was invariably included in the stitches which closed the abdominal wound. All obstinate bleeding points were tied with gut ligature, but the pedicle itself was secured by fine carbolized silk. In three cases where the adhesions were numerous, the glass drainage-tube was employed. The dressing consisted merely of salicylated cotton held in the place by adhesive strips, the whole being secured by an elastic flannel binder. Dr. Goodell prefers the above dry application to Professor Lister's wet dressing. The after-treatment consisted in opium enough to allay pain, and in one tablespoonful of milk combined with lime-water given every three hours for the first forty-eight hours. As soon as flatulence escaped from the bowel the supply of food was increased. The patients were prepared for the operation by a soap-bath on the previous evening, and by the administration of one grain of opium at bed-time. On the following morning one grain of opium and ten grains of quinia were given. Dr. Goodell always operated at eleven o'clock in the morning, as being the time when the vital forces are at th ir best. When high temperature ensued, it was reduced by the application of an ice-cap, which was found to be an efficient means of lowering bodily heat.

**Puerperal Fever** is invariably treated by intra-uterine injections of a warm two per cent. solution of carbolic acid. Ten-grain doses of quinia are given every four hours until marked cinchonism is produced. Morphia is administered in doses sufficiently large and as frequently necessary to relieve pain. The whole surface of the abdomen is painted with the compound tincture of iodine, and covered with a large mush-poultice. If it is deemed necessary to open the bowels, large doses of calomel are used.

**Gathered Breasts.**—When an abscess forms in the mammary glands, an early incision is practiced. If the pus lies deep or is lodged behind the gland, a cutaneous incision is first made, a grooved director is then pushed into the abscess, and the opening is enlarged by the uterine dilator. The breast is then tightly strapped with adhesive plaster, and treated by a dry compress of oakum. Should the abscess show symptoms of becoming chronic, its walls are overdistended by an injection of a three per cent. solution of carbolic acid. This overstretching is practiced so that the acid may reach everv nook and cranny of the purulent cavity.

**The Treatment of the Funis.**—As soon as the child cries lustily the cord is cut, and, the umbilical portion being firmly held by the thumb and forefinger. the free end is "stripped" of Wharton's jelly and of any blood that may remain in it. Any blisters of Wharton's jelly which still remain are emptied by this process of "stripping," are nicked, and their contents squeezed out. After the removal of the pressure of the thumb and forefinger, all bleeding usually ceases, and then the cord is tied. No subsequent dressing is thereafter used, for the cord rapidly dries without smell and drops off without leaving a sore behind.

**Notes on Congenital Syphilis.**—Congenital syphilis appears either before or after birth. The labor is usually premature, and the first symptoms of the disease is the hoarse cry to which the child gives utterance. The bullæ soon show themselves. The disease in utero takes the form of placentitis, the exudation presses the blood out of the small capillaries, and so gradually starves the product of conception; or there may be a gummy tumor or fatty degeneration of the placenta, so causing premature labor. In some cases the labor is precipitated by a theroma of the cord.

He explains the hoarse cry, above referred to, by the presence of syphilitic ulcers on the mucous membrane of the child's throat and air-tubes. Such children are always puny and sickly-looking. The bullæ appear in the course of a few hours after birth, and are first visible on either the scrotum, hands, or feet.

If the disease is not already in existence at birth, it usually begins sometime between the second week and third month after birth. The child cries a great deal at night. This cry, which is occasioned by the incipient bone disease, is muffled and hoarse. Another symptom is the snuffles: the child's nose is all stopped up, and then a scalding coryza comes on. Then the child grows wizened and thin, and its skin lies in rolls and wrinkles. The so-called copper maculæ appear, or the complexion gradually assumes a coffee-and-milk hue. Then the eruption comes out all over the body and stamps the case indisputably as one of syphilis.

**Rhus Aromatica.**—*Enuresis*, arising either from atony of the muscular, or irritation of nervous fibers, will be promptly met by the rhus aromatica. I have relieved many cases in which the patient was unable to restrain the urine to normal distension of the bladder, and others who were unable to prevent constant dribbling of the urine which rendered them filthy and disagreeable, not only to themselves but to those around them, virtually debarring them from society. And if there is a specific for that troublesome condition which we so often meet in children, that of "bed-wetting," we certainly have it in the rhus aromatica. Let one or two illustrations suffice. The mother of John D., aged five years, called January 3d, stating that two years previous her little boy suffered from a severe attack of scarlet fever, and ever since he has been more or less unable to control his urine, and for the past few months he had little or no control over it at all; further, that he would wet the bed two or three times during the night, and himself during the day. She further stated that she had tried several doctors, and almost every remedy that had been suggested to her, for the disagreeable malady, and all without permanent benefit. The case had now become almost alarming and she wanted "something done." I accordingly gave an ounce vial of the first dilution of rhus aromatica and ordered ten drops given three times a day, and to have him empty his bladder before retiring, and to get up immediately on feeling an inclination to urinate. Improvement was rapid; at the expiration of six weeks the morning and noon doses were discontinued, dose at night continued; at the end of three months I pronounced my patient cured.—*Eclectic Medical Journal.*

**Electricity in Amenorrhœa.**—Electricity is applicable to the treatment of many cases of amenorrhœa. Dr. Golding-Bird expresses his belief that it is the only direct emmenagogue we possess, and that it always excites menstruation where the uterus is capable of performing that function. Electricity is especially valuable as an emmenagogue in young women, where the menstrual function has not yet been fully established in consequence of a torpid state of the vaso-motor nerves of the ovaries and uterus; and also when the catamenia have been suppressed after labor, or in consequence of a chill or emotion. Faradization of the womb has been practiced with good results. Electricity may be applied also to those cases of defective involution of the uterus, in which this organ is enlarged and impotence the result. Galvanic pessaries have also been used with good results in such cases. Dr. G. Murray succeeded by the introduction of the galvanic pessary in reducing in the course of the fortnight, the large and flabby uterus to its normal and healthy condition.—*Ibid.*

**Danger in the Use of Podophyllin.**—A correspondent of the British Medical Journal describes four cases of injury resulting from podophyllin, the dose taken being named in only one case, when one-fourth of a grain, taken night and morning, three times, caused colicky pains and tenesmus for two days. It would be well to remember, adds the writer, that when patients are bothered with their bowels it is not the purgative effects of a medicine that are generally required, but the alterative; so that a half, or may be a quarter, grain of calomel, or one-sixth of a grain of podophyllin, night and morning, will frequently—indeed, almost invariably—produce more beneficial and lasting results than the larger doses..

In a subsequent number of the same journal, Dr. Horace Dobell says: "All who have been accustomed to prescribe podophyllin in pills will agree as to the impossibility of preventing occasional disastrous effects. But this is the fault of the form of administration, not of the drug. From a very long and extensive experience I can confidently say that no such accidents or inconveniences ever arise from podophyllin prescribed in the following form; on the contrary, it is one of the most satisfactory and reliable of our medicines:

℞ Podophylli........................gr. ij.

Essentiæ zingiberis..... ....ʒ ij.

Spiritus vini rect.................ad ℥ ij.

Fiat guttæ. A teaspoonful to be taken in a wineglassful of water at bedtime every night, or every second, third, or fourth night, as required. Numbers of medical men, to whom I have given this prescription within the last few years, tell me that they use it in their daily practice with the happiest results."—*Boston Journal Medicine.*

**Electricity in Impotence.**—In regard to the mode of applying this agent, a word may be said. According to Benedict, we should place the copper pole of the constant battery over the lumbar vertebræ, and pass the zinc pole, forty or fifty times, in the direction of the spermatic cord; then transversely over the different zones of the upper and lower surfaces of the thighs, and then, likewise, in the perineum. The sittings should last two or three minutes, about three times a fortnight. The copper pole should be applied by means of a catheter-shaped sound to the vicinity of the ejaculatory duct, and passes should be made with the zinc pole in the direction of the spermatic cord, if there are any particularly insensitive places. Benedict uses Faraday's galvanic brush; and if the testicles are peculiarly insensible, he passes a strong current through them. The sittings should take place every day, and should be continued for some time, as improvement does not take place for months in some cases.

Schultz, in Vienna, has, for a long time, used the induced current for pollutions and impotence. Under this treatment, the success was very poor, but he claims that it is greater since he has commenced using the constant current. He places the positive pole over the fifth dorsal vertebræ; the negative over the sacrum, or on the perinuem. The sitting should last from one to three minutes, to be repeated three or fonr times a week. Schultz employs a battery with twenty or thirty Stohrer elements of medium size.—*Dr. Caldwell in Obstetric Gazette.*

**Intra-Uterine Medication.**—In a paper read by Dr. James P. White, of Buffalo, New York, before the American Gynecological Society, on the subject of intra-uterine medication, he passed over pathological conditions and the therapeutics of those conditions, and simply gave some hints regarding the means which had been found available in the proper application of remedies to the mucous membrane of the neck and body of the uterus. Although intra-uterine injections were advisable by some, they were rarely resorted to by the experienced practitioner. He spoke of the necessity of enlarging the cervical canal before resorting to this method of treatment. He recommended shallow incisions into the mucous membrane of the neck of the uterus, to facilitate in certain cases the introduction of sponge-tents. If properly made, no harm followed the incisions. After removing the catarrhal discharge from the cervix, he very commonly used the following as a local application:

℞ Iodine.................................................ʒj.

Iodide of potassium......................ʒss.

Tannin.............................................ʒi.

Glycerine........................q. s. to dissolve.

M.

Dr. White then exhibited several instruments to be used in carrying out intra-uterine medication.—*Obstetric Gazette.*

**Plugging the Cervix Uteri for Metrorrhagia.**—At a late meeting of the Obstetrical Society of Paris, a discussion on the treatment of metrorrhagia was introduced, and among the various manipulative measures that were referred to, preference was given to plugging the cavity of the neck of the womb, which had several advantages over plugging the vagina in such cases. It stopped the blood more effectually, the patients bore it better, and there was less chance of putrid absorption. Each speaker recommended his own plan, but that adopted by M. Panas seems to me the best. It consists of introducing into the cavity of the uterine neck a pledget of cotton wool, rolled up to about the thickness of a goose-quill, and steeped in a solution of the perchloride of iron of the Cordex, to which is added one part of water, to prevent its caustic effects. This being done, he introduces a ball of cotton wool and places it in the posterior cul-de-sac of the vagina, where it not only forms a support to the uterine plug, but it absorbs any liquid that may escape through it, and thus protects part of the vagina (which is covered with the peritoneum) from the corroding effects of the perchloride of iron and the acrid discharges from the womb.—*Medical and Surgical Reporter.*

**The Liquor Amnii.**—According to Dr. H. Fehling, the liquor amnii about a mature child amounts to one-half to three-quarters liter, and it increases in the last 4—6 weeks of pregnancy 200—250 grammes.

The development of the fetus and the quantity of the liquor amnii stand in no demonstrable relation, as little does the sex of the fetus. on the other hand, length and torsion of the umbilical cord exercises a certain influence upon the quantity of the liquor amnii. With an increase of the spirals of the cord there is an increase in the quantity of the waters, as there is also in deep insertion of the cord into the placenta in consequence of increased pressure of the waters.

That urine is mixed with the liquor amnii seems from Gusserom's investigations beyond doubt. The fetus urinates regularly and this urine is a chief constituent of the liquor amnii. To ascertain how much the fetus urinates in a given time, Fehling endeavored to discover how much foreign matter entered the liquor amnii. For this purpose he administered to pregnant women daily doses of salicylate of soda and ferro-cyanide of potash. As the conditions for exact date are difficult of attainment, but a few experiments could be made. In three experiments with salicylic acid, one was negative, one showed that 40 grammes of urine was mixed with one liter of liquor amnii in twenty-six days, and the third that about 65 cubic centimetres of urine was emptied into 655 cubic centimetres liquor amnii. In seventeen experiments with the ferro-cyanide of potash this agent could be demonstrated in the liquor amnii but three times. But it must not be forgotten that these materials foreign to the circulation could reach the liquor amnii through other avenues than the kidneys, as through the skin, cords, envelopes, etc. Fehling shows indeed that exosmosis exists in the cord, but his experiments are not yet couclusive. That the infantile kidney does not act like that of the adult, is shown by a series of experiments. The kidneys of the mother do not excrete the salicylate of soda half as fast as those of the child.—*Centralblatt f. d. Med. Wisenschaft*, August 19, 1879.

**Acute Nephritis.**—J. H., æt. 29. Three weeks ago, after exposure to cold, was taken with rigor, followed by fever, pain in region of kidneys, and violent emesis. Had constant desire to urinate; able to pass but few drops at a time. At present, face and limbs greatly swollen (œdematous); urine small in quantity, light in color; sp. gr. 1009, by heat and acid coagulates one-half; contains epithelium from tubes and passages, and epithelial and bloody casts. Lungs œdematous. At 7 in the evening, was given 15 minims fld. ext. jamborandi; at 8 in the evening, a second dose; soon after perspired freely, and saliva escaped continuously. Following day, better; jamborandi repeated in the evening, with dry cups over kidneys and lungs; third day, the same; fourth day, œdema disappeared; stopped jamborandi. Sixth and seventh days, slight œdema; jamborandi repeated in fifteen and sixty minim doses, but no perspiration produced—only salivation. Urine, sp. gr. 1012; albumen, one-fourth. Ordered mixture of tr. digital, Pot cit., and Buchu. Four days after, discharged; entirely recovered.—*Medical and Surgical Repoter*.

**Injections of Water in Dysentery.**—Dr. Shaw (St. Louis Medical Journal) reports a case of dysentery thus: Three hours after the preceding visit, we saw her again, and concluded to desist from any further use of opium, to introduce the largest possible quantity of warm water, slightly tinctured with common salt, into the bowel with common salt three and a half quarts were injected at the third trial), and give beef essence and whisky by the mouth. In seven or eight hours the tenesmus and tormina had almost entirely ceased.

After twenty-four hours of this treatment the temperature of the injections was gradually reduced, until in eighteen hours more, the water as it flowed from the hydrant was used. These cold water enemas were continued until the 11th, when an interval of four hours between injections was ordered, and the salt was omitted.

By this time the character of the stools had so changed that they were no longer dysenteric, but diarrhœal; the tenderness so particuly marked in the region of the ilio-cæcal valve and all along the course of the colon, had entirely disappeared, and the appetite was craving.

On the 16th, the lady was able to sit in a chair for an hour, without any great sense of fatigue. The enemas having been omitted during the night, the fæces passed the next morning were shaped, and quite natural in color.

From this time to the 21st, iujectibns were made every twenty-six hours during the day, and omitted at night. On the 21st, the enemas were discotinuued. About one week subsequently, after a rather free indulgence in apples and plums, there was a return of dysenteric stools, but they were rendered diarrhœal in twelve hours, by enemas of cold water, repeated every two hours. Under their contiuuance this relapse was completely relieved in thirty-six hours.

**Treatment of Dysentery by Injections of a Solution of Chloral.**—In the case of a child of eleven, who suffered with thirst, pain, tenesmus, and had twenty-five to thirty dejections in twenty-four hours, 5 gr. of chloral hydrate in 2 oz. of starch gruel were thrown up the bowels with considerable force, from a hard rubber syringe. The injection remained three hours, during which time the child slept. Many of the other symptoms were modified, and the injection was repeated, remaining seven hours, and then came away with some fœcal matter, but without tenesmus. Four enemas were given, in all, and treatment was discontinued in forty-eight hours. It was also tried in the case of a lady, aged twenty-five, using ten grains instead of five, and securing complete repose for eight hours, although she had previously had twenty to thirty movements in twenty-four consecutive hours. In conclusion: "The number of aggravated cases of dysentery we have treated with chloral hydrate warrants us in the assertion that, if early and promptly used, it is almost a specific.—Dr. Newell, in the *Medical Times*.

**Atropia in Croup.**—The sulphate of atropia is a one per cent. solution, and has been used with success in the treatment of a case of croup which threatened a fatal termination. Dr. De Poutenes, of Antibes, reports in *L' Union Medicale*, that in the third day of the attack, death seemed inevitable, medication having failed of benefit; the epigastrium was retracted, the face and neck swollen and purple; three drops of the solution was injected on the left side of the neck, on a level with the pneumogastric; although there was in a few minutes improvement, in four hours the dose was repeated, amelioration was decided, and in a few days recovery was complete. The suggestion is a valuable one, in such cases as are threatening suffocation from other causes than exudation, and which are accompanied by congestion and œdema, resulting perhaps from, or associated with, more or less paralysis of the pneumogastric, to which nerve, belladonna is a special excitant.—*American Journal Medical Science, and Dublin Journal Medical Science*, January, 1879.

**Moral Dietetics.**—Dr. Bock, of Leipzig, writes as follows on the moral effect of different articles of food:

The nervousness and peevishness of our times are chiefly attributable to tea and coffee; the digestive organs of confirmed coffee drinkers are in a state of chronic derangement, which reacts on the brain, producing fretful and lachrymose moods. Fine ladies addicted to strong coffee have a characteristic temper, which I might describe as a mania for acting the persecuted saint. Chocolate is neutral in its psychic effects, and is really the most harmless of our fashionable drinks. The snappish, petulant humor of the Chinese can certainly be ascribed to their immodest fondness for tea. Beer is brutalizing, wine impassions, whisky infuriates, but eventually unmans. Alcoholic drinks, combined with a flesh and fat diet, totally subjugate the moral man, unless their influence be counteracted by violent exercise. But with sedentary habits, they produce those unhappy flesh sponges which may be studied in metropolitan bachelor halls, but better yet, in wealthy convents. The soul that may still linger in a fat Austrian is about as functional to his body as salt is to pork—to prevent imminent putrefaction.—*Cin. Clinic and Lancet.*

**Notes from Practice.**—The following notes may be useful to practitioners:

1. A drop of glycerine allowed to fall into the hypodermic syringe will keep the piston moist for an indefinite time, and prevent the annoying delay which the physician is apt to experience when he wishes to use his hypodermic syringe after a few days' rest.

2. The tin bellows which is manufactured for the purpose of blowing the poison into the holes where roaches may be lurking, forms a more convenient, and surely safer, method of applying sulphur locally to the throat in diphtheria than a quill, as has been recommended.

3. Many physicians wish to preserve permanently the tracings made on the glass by the sphygmograph. The following method is, I believe, original:

Make a strong solution of the ferrocyanide of potassium, (the red, not yellow, prussiate of potash). Paint this solution over some sheets of writing paper, allow the paper to dry in the dark, and keep them secluded from the light. When it is required to make a copy of the tracings, cut a piece of the paper of an appropriate size, and having laid the glass upon it, face downward, expose it for some hours to the sunlight. Then remove the paper, and wash it in clean water. The sphygmographic curve will be found permanently printed in blue.—*Dr. Kelly, in Medical and Surgical Reporter.*

**A Case of Amenorrhœa.**—The Medical Press and Circular gives the following case from the clinic of Dr. Lyster, of Liverpool:

C. Q., aged twenty-two, came under my notice suffering from great pain over the left eye and temple, loss of appetite and debility.

*History.*—The catamenia commenced at the age of thirteen, and were regular till twelve months ago, when they ceased. Every kind of medical treatment was tried, without avail, while the patient was suffering great pain, and becoming thin and anæmic.

On examination per vaginam, the cervix uteri was found thickened, elongated, and very tender to the touch, and from the os uteri was hanging a thick, gelatinous secretion. As a primary measure the cervix was punctured and relieved of a few ounces of blood, and a glycerine pad placed against the os uteri. Relief was obtained, but the pain and discomfort returning, it was thought advisable to dilate the cervix with sea-tangle, and cauterize the interior of the uterus with carbolic acid. Glycerine pads applied to the uterus till the sloughs separated. The pains ceased, the appetite returned, and the patient was discharged from the hospital; but the catamenia not returning, the patient again applied for admission. A galvanic pessary was introduced, which brought on the catamenia in a week; menstruration was again deficient, but the introduction of the galvanic pessary was again sufficient to produce them, and since then the patient has been in good health and menstruates freely.—*Medical Reporter.*

**Narcotism from Nutmeg.**—Mrs. N., aged thirty-eight, mother of four children, was confined on Sabbath morning, June 29, 1879, at nine o'clock. The child was a girl, and the largest I ever saw; weight fourteen and one half pounds. Labor natural and easy; had a spasm after the last pain; the spasm was hysterical. On the 30th the "old woman" persuaded her to take nutmeg tea. One and a half nutmegs were used in making the tea, and she drank it during the day. About 10 p. m. she began to get drowsy. By four o'clock next morning she was in a profound stupor. At ten a. m. the narcotic effects of the nutmeg began to die out, and by four p. m. she had pretty well recovered. The symptoms were about the same as those produced by opium, and the remedies were the same. I mention this case for the reason that nutmegs are in such general use as a condiment that we may lose sight of their dangerous narcotic tendencies. In twenty-one years' practice I have never seen such a case before, and if I had ever known that the nutmeg possessed such properties it had completely escaped my memory, and for fear that some of our numerous professional brethren may be in a like condition, I have deemed it proper to mention this case.—*Dr. H. Barry in St. Louis Clinical Record.*

**Sanitas, the New Disinfectant.**—We have given this new disinfectant a sufficiently extensive trial to be satisfied that it possesses great merits, and is worthy of a place beside the most popular disinfecting agents now in use. Granting that its disinfecting powers are equal to those of other preparations, it has the great advantage of a pleasant odor, of not being a poison, and of not injuring clothing, furniture, etc., with which it is brought in contact. There is no secret about its manufacture. Russian turpentine and water are placed in huge earthenware jars, surrounded by hot water. Air is driven through the mixture in the jars continually for three hundred hours, the result being a decomposition of the turpentine, and the formation of a watery solution of the substance, to which Dr. Kingsett, the discover, has given the name of "Sanitas." After evaporation, the substance, as sold in tin cans, is a light brown powder, of a pleasant taste and odor, and capable in a very remarkable degree of preventing or arresting putrefactive changes. It has been in use for some time in England, and is now being introduced here.—*New York Medical Journal.*

**Boldo** acts as a stimulant to digestion, and exerts a marked influence on the liver; this property residing in both the leaves and young stems. A flock of sheep ate the boldo twigs, causing them to pass large quantities of the "liver fluke," or gourd worm, which produced the so-called liver disease. Hence the discovery of their medicinal qualities.—*New Remedies.*

**Singular Conflict of Opinion.**—A writer in an Eastern journal says: "A prolific source of puerperal fever, in my opinion, is the administration of opiates for the relief of after-pains." Many obstetricians regard opiates in the opposite light. The present writer has employed them in labor almost invariably for many years, for the purpose of mitigating pain and relaxing the os uteri, the indication being the same as that leading to the use of chloroform. Thus exhibited they not only expedite delivery, but prevent after-pains. And so far from inducing puerperal fever, long experience has established the conviction that this treatment protects the patient from it. We have never hesitated to give opiates, mostly in combination with camphor, for the relief of after-pains. We know a number of others who pursue the same course with the same favorable results. There is no point in obstetric practice more firmly established in our mind than that opiates during labor, and subsequently if necessary, are a safeguard against puerperal fever.—*Eclectic Medical Journal.*

**Rag Weed as a Remedy for Rhus Poisoning.**—J. A. Zabriskie writes to New Remedies as follows: Having read your valuable journal for nearly two years, I have at various times seen articles relating to the value of different drugs in their action on the poison of Rhus, and, among others, that of Grindelia robusta, which is claimed, especially on the Pacific coast, to be a specific. I would like to add one more to the list, which I consider equally as efficacious, and it has the advantage of being always obtainable.

The one I mean is Ambrosia artemisiœfolia, or rag weed. The mode of applying it is this: Take the fresh leaves, any convenient quantity, bruise them, and apply the juice that exudes from them to the surface of the parts affected with the poison, rubbing until the skin is discolored, when almost instant relief will be felt.—*Medical Summary.*

**Infusion of Buckeye as a Remedy for Chronic Rheumatism.**—Dr. W. S. Drake had an inveterate case of chronic rheumatism cured by the patient bathing in an infusion of buckeye (esculus hippocastonum). The patient had not walked for nearly two years, and had gone through the whole routine of rheumatic remedies. While treating a horse with infusion of buckeye, he found the swelling rapidly disappeared from his hands. He then applied it to other joints, and received the same benefits.—*St. Louis Medical and Surgical Jonrnal.*

**Ice Cream and Beef Juice.**—As an excellent dietary article, this is praised by Dr. J. J. Tucker, in the Chicago Journal. His formula is: R. Cream, 120 grams; sugar, 30 grams; extract of vanilla, 8 grams; beef juice, 8 grams. Any confectioner can make it, or it may be readily prepared at home with a freezer. Its uses are obvious.—*Hospital Gazette.*

**Membranous Dysmenorrhœa.**—Dr. Barker, in Gynecological Society, relates three cases of membranous dysmenorrhœa, which he has cured by means of intra-uterine applications of iodoform in the form of cones. First dilate the cervix, then introduce cones, one on every other day. He showed a syringe to be used as an applicator.

**The First Insensibility from Ether.**—For the short operations of minor surgery, and the reductions of dislocations, or opening of abscesses, it is extremely useful and of every-day application. Such a patient wishes to be operated upon wtthout pain, or from being incapacitated from attending to business during the remainder of the day. He lies down upon the sofa, and with one hand places the ether inhaler, on a sponge wet with ether, over his face, mouth and nose, and holds the other arm and hand up in the air.

This arm, after the ether has been breathed for a few minutes, will drop, and from thirty to fifty seconds of unconsciousnoss will be had, in which to operate. The sponge being removed, the patient is ready to go about his business. It gives rise to no headache, nausea, or other unpleasant symptoms, and is particularly useful in children. The chief source of disappointment is in not recognizing the right moment, for if this is allowed to pass, unconsciousness will not occur until full etherization. The first insensibility is sure to come. When the arm moves, be ready, and as soon as it drops perform the operation; no pain will be felt.—*Medical Times.*

**Eucalyptus in Bronchitis.**—Dr. Bell says: "The eucalyptus globulus has remarkable anti-catarrhal virtues. The only preparation which I have used has been the tincture prepared by several of our most eminent druggists in Edinburgh, and I have seldom prescribed more than a teaspoonful mixed with a wineglassful of water twice a day. In several cases of bronchitis, with profuse expectoration, I have witnessed remarkable benefit after a very brief use of the remedy, evinced by a rapid diminution of the discharge, and also by a corresponding improvement in the general condition of the patient."—*Druggists' Circular.*

**Kolpoecpetasis Versus Kolpokleisis, as Illustrated in a Case of Artesia of the Vagina with Recto-Utero-Vaginal Fistule.**—The above is the title of a paper presented by Dr. Nathan Bozeman, of New York, which would have been read had the time permitted. He used the word kolopoecpetasis (to stretch the vagina) in contradistinction to the word kolpokleisis (to close the vagina). The case reported illustrated the meaning of both terms.

[We copy this to protest against the coining of so many jaw-breaking technicalities. The above strikes us as exceedingly far-fetched and unnecessary.—ED.]

A GARGLE of great service in commencing angina, and in all chronic irritations of the throat, is prepared with carbolic acid and tannin, of each fifteen parts, alcohol sixty parts, distilled water one hundred and twenty parts. A teaspoonful in half a glass of water. Use once, or oftener, daily.

IN the vomiting of pregnancy pure carbolic acid when applied to the cervix uteri is just as efficacious as stronger caustics, and is better for the organ in question.—*Boston Medical and Surgical Journal.*

